# Hospital readmissions in the group of users on the Flexible Assertive Community Treatment – experiences from RECOVER E Montenegro samples

**DOI:** 10.1192/j.eurpsy.2022.800

**Published:** 2022-09-01

**Authors:** J. Dedovic, T. Djurisic, A. Tomcuk, A. Macic, N. Matkovic, D. Miladinovic, S. Vlahovic

**Affiliations:** 1 Special Psychiatric Hospital Kotor, Department For Forensic Psychiatry, Kotor, Montenegro; 2 Public Health Institute of Montenegro, Health Promotion Center, Podgorica, Montenegro; 3 Special Psychiatric Hospital Kotor, Department For Mental Health Promotion, Kotor, Montenegro; 4 Special Psychiatric Hospital Kotor, Chief Executive Office, Kotor, Montenegro; 5 Special Psychiatric Hospital Kotor, Addiction Department, Kotor, Montenegro; 6 Special Psychiatric Hospital Kotor, Acute Male Ward, Kotor, Montenegro

**Keywords:** Hospital readmissions, Community Mental Health Teams, Flexible Assertive Community Treatment, RECOVER E, Horizon 2020

## Abstract

**Introduction:**

As a part of Horizon 2020 program, RECOVER-E project activities were initiated in Montenegro in 2018. The initial step involved a thorough situation analysis of the setting and circumstances of treatment of users with severe mental health illnesses, followed by the establishment of the community mental health team (CMHT) within the Special Psychiatric Hospital Kotor. The CMHT became responsible for the treatment of a group of clients with severe mental health illnesses, based on the principles of „Flexible Assertive Community Treatment (FACT – A Dutch model).

**Objectives:**

The main objective of this research was to establish whether there were substantial differences regarding the hospital readmissions in the group of patients treated by the CMHT, compared to usual mental health care in Montenegro.

**Methods:**

Within the RECOVER-E project, a sample of 202 patients, users of mental health services, were recruited in Montenegro. Patients were randomized into two similar-sized groups - intervention group, whose treatment was managed by the multidisciplinary CMHT, and control group where treatment as usual was continued. To estimate and follow-up the frequency of hospital readmissions, medical documentation was used.

**Results:**

Patients in the intervention group had less hospital days during the 18 months follow-up period. However, the differences between two groups regarding number of readmissions, and total length of hospital days were not statistically significant measured by independent T test.

**Conclusions:**

This study showed that CMHT care could reduce the total length of hospital days during the treatment of psychotic disorders even dough during the COVID 19 pandemic and lock down measures

**Disclosure:**

No significant relationships.

